# Expanding the horizon of sex‐specific proteomic insights in Alzheimer's disease

**DOI:** 10.1002/alz.70725

**Published:** 2025-09-22

**Authors:** Yue Zhou, Shuang Liu, Lizhi Chen

**Affiliations:** ^1^ Department of Science and Education the Affiliated Guangdong Second Provincial General Hospital of Jinan University Guangzhou China; ^2^ Department of Emergency Guangzhou Tianhe District Central Hospital Guangzhou Dongsheng Hospital Guangzhou China

1

Dear Editor,

The recent study by Mei et al. provides a pivotal contribution to understanding sex differences in Alzheimer's disease (AD) through comprehensive proteomic profiling of the dorsolateral prefrontal cortex (DLPFC).^1^ By identifying 10 proteins with sex‐specific interactions in AD pathogenesis, cognitive trajectories, and cerebral pathologies, this work advances the field beyond transcriptomic analyses and highlights the importance of sex‐aware biomarker discovery. We commend the authors for their rigorous approach and propose additional perspectives to further contextualize these findings within the broader landscape of AD research.

First, the identification of sex‐biased proteins linked to estrogen signaling (e.g., PLCD3, SLC22A5) aligns with emerging evidence that estrogen receptor activity modulates synaptic plasticity and neuroinflammation in a sex‐dependent manner.^2^ For instance, G protein‐coupled estrogen receptor (GPER) activation enhances phospholipase C signaling, potentially influencing amyloid beta clearance.^3^ These findings resonate with epidemiological studies showing that estrogen depletion in postmenopausal women accelerates AD‐related cognitive decline.^4^ Further investigation into how estrogen‐responsive proteins interact with apolipoprotein E genotype, a known modifier of AD risk, could clarify why females exhibit greater resilience to early amyloid deposition despite higher lifetime AD incidence.^5^

Second, the association of S100A12 (a pro‐inflammatory mediator) and MTFR1L (a mitochondrial fusion regulator) with sex‐specific cognitive decline underscores the interplay between neuroinflammation and bioenergetic dysfunction in AD. Elevated S100A12 colocalizes with amyloid plaques and correlates with microglial activation in AD brains,^6^ and neuroinflammatory responses are more pronounced in females with AD neuropathology.^7^ Meanwhile, mitochondrial dynamics are critical for maintaining neuronal connectivity, and sex differences in mitochondrial gene expression have been reported in aging brains.^8^ Integrating these proteomic results with metabolomic or single‐cell transcriptomic datasets may reveal how inflammatory and metabolic pathways converge to drive sex‐divergent disease trajectories.

Third, the study's focus on the DLPFC, a region affected relatively late in AD, raises intriguing questions about whether sex‐specific proteomic signatures emerge earlier in vulnerable regions like the entorhinal cortex or hippocampus. Prior work has shown that tau propagation follows distinct spatial patterns in males and females, with females exhibiting faster neocortical spread independent of amyloid burden.^9^ Validating the 10 candidate proteins in preclinical cohorts or fluid biomarkers (e.g., cerebrospinal fluid, plasma) could determine their utility for predicting sex‐specific rates of clinical progression.

Finally, while the homogeneity of the cohort (European ancestry) ensures methodological rigor, future studies should assess whether these proteomic signatures generalize across diverse populations. For example, ancestry‐related differences in immune response and vascular risk factors may modulate sex effects on AD pathogenesis.^10^ Extending this work to multi‐ethnic cohorts, such as the Alzheimer's Disease Neuroimaging Initiative (ADNI) or Health and Retirement Study (HRS), would strengthen the translational relevance of sex‐specific biomarkers.

In summary, Mei et al. have provided a foundational framework for exploring sex differences in AD at the proteomic level.^1^ Their findings open new avenues for developing personalized therapeutic strategies and underscore the necessity of considering sex as a biological variable in both preclinical and clinical AD research.

## AUTHOR CONTRIBUTIONS

*Conceptualization*: Yue Zhou. Study design: Shuang Liu. *Manuscript draft*: Yue Zhou. *Draft review and editing*: Lizhi Chen.

## CONFLICTS OF INTEREST STATEMENT

The authors declare that they have no competing interests. Author disclosures are available in the Supporting Information.

## FUNDING INFORMATION

This work was funded by the National Natural Science Foundation of China (Nos. 82204791), Natural Science Foundation of Guangdong Province (Nos. 2020A1515010002), Science and Technology Projects in Guangzhou (Nos. 2025A04J5526).

## ETHICS APPROVAL AND CONSENT TO PARTICIPATE

Not applicable.

## CONSENT FOR PUBLICATION

The authors have seen and approved the final manuscript.

## Supporting information



Supporting information

## Data Availability

Not applicable.
